# Simplified MethylRAD Sequencing to Detect Changes in DNA Methylation at Enhancer Elements in Differentiating Embryonic Stem Cells

**DOI:** 10.3390/epigenomes4040024

**Published:** 2020-10-01

**Authors:** Debapriya Saha, Allison B. Norvil, Nadia A. Lanman, Humaira Gowher

**Affiliations:** 1 Department of Biochemistry, Purdue University, West Lafayette, IN 47907, USA; 2 Purdue University Center for Cancer Research, Purdue University, West Lafayette, IN 47907, USA; 3 Department of Comparative Pathobiology, Purdue University, West Lafayette, IN 47907, USA

**Keywords:** DNA methylation, embryonic stem cells, MethylRAD, enhancer, pluripotency genes, histone demethylase LSD1

## Abstract

Differential DNA methylation is characteristic of gene regulatory regions, such as enhancers, which mostly constitute low or intermediate CpG content in their DNA sequence. Consequently, quantification of changes in DNA methylation at these sites is challenging. Given that DNA methylation across most of the mammalian genome is maintained, the use of genome-wide bisulfite sequencing to measure fractional changes in DNA methylation at specific sites is an overexertion which is both expensive and cumbersome. Here, we developed a MethylRAD technique with an improved experimental plan and bioinformatic analysis tool to examine regional DNA methylation changes in embryonic stem cells (ESCs) during differentiation. The transcriptional silencing of pluripotency genes (PpGs) during ESC differentiation is accompanied by PpG enhancer (PpGe) silencing mediated by the demethylation of H3K4me1 by LSD1. Our MethylRAD data show that in the presence of LSD1 inhibitor, a significant fraction of LSD1-bound PpGe fails to gain DNA methylation. We further show that this effect is mostly observed in PpGes with low/intermediate CpG content. Underscoring the sensitivity and accuracy of MethylRAD sequencing, our study demonstrates that this method can detect small changes in DNA methylation in regulatory regions, including those with low/intermediate CpG content, thus asserting its use as a method of choice for diagnostic purposes.

## 1. Introduction

DNA methylation is an epigenetic modification that plays an important role in many biological processes like genomic imprinting, X chromosome inactivation, embryogenesis, cellular differentiation, and transposon silencing [1]. In mammals, the DNA methyltransferases (DNMTs) DNMT3A and DNMT3B establish DNA methylation during early embryogenesis [2] and the post-replication maintenance of DNA methylation is largely performed by the enzyme DNMT1 [3,4]. DNA methylation involves the transfer of a methyl group from S-adenosyl methionine to the cytosine base mostly in CpG dinucleotides of the DNA [1,5]. The CpG dinucleotide is underrepresented in the mammalian genome [6,7] except in regions distinctly identified as CpG islands, which tend to be largely unmethylated [8]. CpG islands are found overlapping with the promoters of constitutively expressed house-keeping genes and are also associated with regulatory regions of imprinted genes. The promoters of tissue-specific genes and distal regulatory elements, such as enhancers, usually constitute low/intermediate CpG content and are differentially methylated [9]. The methylated state of promoters and enhancers is usually associated with gene repression. An increasing body of evidence suggests that the methylation status of enhancer regions corresponds better with target gene expression compared to the promoter methylation [10]. Given the that the DNA methylation state of the enhancer can be a direct readout of the transcription state, the aberrant DNA methylation of enhancers may be an indicator of a diseased state [11]. Mutations in DNMTs causing DNA methylation changes are highly prevalent in diverse pathological manifestations, such as neurodegeneration, growth syndromes, hematological malignancies, etc. [12].

Given that DNA methylation changes occur only in a fraction of the genome and these changes have a strong regulatory potential, there is a need for the development of high-throughput approaches to specifically and accurately measure changes in DNA methylation at these sites. Whole genome bisulfite sequencing (WGBS) uses bisulfite conversion of unmethylated cytosine residues to uracil and provides methylation information at a single-base resolution of the whole genome [13,14]. Since the bulk methylation across the genome is maintained, the generation of huge datasets to measure changes in only a fraction of the genome is futile. Some limitations of WGBS are partly alleviated by reduced representation bisulfite sequencing (RRBS), which is based on bisulfite sequencing of size-selected DNA fragments generated by restriction digestion. However, mapping the RRBS data to a reference genome can be challenging because bisulfite conversion reduces the genome complexity and the sequence alignment of short fragments becomes difficult [15]. Moreover, in WGBS, the measured methylation in differentially methylated regions is highly affected by biases caused by bisulfite conversion and other experimental variables, supporting the development of techniques which use bulsulfite-independent measurement of methylation in those regions [16]. Immunoprecipitation-based methods like Methylated DNA immunoprecipitation (MeDIP) utilize the pull-down of methylated DNA using anti-methylcytosine-binding proteins (MBDs) or antibodies against 5mC. The limitations for these techniques include low resolution and bias towards CpG-rich and highly methylated regions [17–20]. Methylation-dependent restriction enzymes serve as unique tools for the identification of methylated bases [21]. FspEI is a type IIS Mrr-like enzyme that recognizes 5-methylcytosine (5mC) and 5-hydroxymethylcytosine (5hmC) in the C^m^C and ^m^CDS sites (D = A or T; S = C or G) [22]. FspEI generates a double-stranded cleavage on the 3′ end of the modified cytosine at a fixed distance (N_12_/N_16_), cutting bi-directionally to generate 32 base-pair fragments in the presence of symmetrically methylated target sites. When combined with next generation sequencing (NGS), this method, known as MethylRAD, is a powerful tool for global methylome profiling. The conventional MethylRAD technique involves the retrieval of specific DNA fragments from polyacrylamide gel before library construction [23], making it technically challenging and frequently irreproducible.

In this study, we modified the MethylRAD technique and tested the efficacy and sensitivity of the method by analyzing changes in DNA methylation in the low/intermediate CpG content regions, specifically the enhancers of pluripotency genes during embryonic stem cell (ESC) differentiation. ESCs possess a unique DNA methylation signature with a specific methylation pattern that is very distinct from differentiated cells or cancer cells [24–28]. The undifferentiated state of ESCs is marked by the high expression of pluripotency genes. Consequently, the distant regulatory elements of pluripotency genes (PpGs), the pluripotency gene enhancers (PpGes), are in an active state, which is characterized by the binding of transcriptional coactivators, the presence of unmethylated DNA, and chromatin modifications, including histone H3K4me2/1 and H3K27Ac, at these sites. In response to signals of differentiation, the coactivator complex dissociates from PpGe, and concomitantly the LSD1-Mi2/NuRD complex, with its histone deacetylase (HDAC) activity, removes H3K27Ac, and the histone demethylase LSD1 demethylates H3K4me1 [29]. In our previous studies, we showed that histone demethylation by LSD1 is required for the subsequent deposition of DNA methylation in PpGes [30]. However, these observations were made only in a subset of seven PpG enhancers. Using the improvised MethyRAD technique, we investigated the genome-wide DNA methylation in ESCs before and after differentiation and measured the changes in DNA methylation at all known PpGes. To confirm the role of LSD1 in the regulation of DNA methylation in PpGes, ESCs were treated with an LSD1 inhibitor, pargyline, for 6 h before the induction of differentiation and DNA methylation in PpGes was compared between the treated and untreated samples. Our data show a significant increase in methylation in ~27–29% of LSD1-bound and decommissioned PpGes post differentiation, which was affected significantly by pargyline treatment. Our data therefore confirm the role of LSD1 in regulating DNA methylation and show its widespread effect on PpGe silencing during ESC differentiation. Our study also emphasizes the use of MethylRAD as a simple, sensitive, and largely accurate high-throughput method to measure DNA methylation changes in regulatory regions, such as enhancers and other regions with low/intermediate CpG content, without the use of chemical modification. The technical ease and simpler analysis of the datasets generated by this method makes it a powerful tool to analyze methylation changes at specific sites in various diseased states.

## 2. Results

### 2.1. Analysis of DNA Methylation at FspEI Sites during ESC Differentiation

During embryonic development, genome-wide changes in gene expression are associated with the site-specific gain of DNA methylation in regulatory regions. DNA methylation is established by the de novo DNA methyltransferases DNMT3A and DNMT3B [2,31]. Our previous studies have shown in vitro that during the differentiation of mouse embryonic stem cells [ESCs], the deposition of DNA methylation by DNMT3A is disrupted by the inhibition of the histone demethylation by LSD1 in a subset of pluripotency gene enhancers (PpGes) [30]. To determine if the gain of DNA methylation was widespread in other known PpGes and was regulated by LSD1 activity, we differentiated ESCs for 7 days in the presence or absence of the LSD1 inhibitor, pargyline. DNA methylation of the three samples [undifferentiated, untreated, and treated differentiated] was measured using our improved MethylRAD sequencing technique.

MethylRAD sequencing uses the methylation-dependent restriction enzyme FspEI, which cuts at 10–16 bps flanking the methylated cytosine. This results in a release of 30–35 bp fragments, which are isolated and sequenced to determine methylated regions in the genome. However, the conventional method involves a tedious and frequently irreproducible step of DNA extraction for library preparation. We simplified the method, by gel excising the 30–35 bp fragments after the digested DNA was separated on an agarose gel. This pool of fragments was used for library preparation and sequencing (Figure S1A,B). Sequencing generated a total of 176.9 million reads. Individual sample reads were first mapped to the reference sequence mm10 to identify C^m^CGG/C^m^CWGG containing FspEI recognition sites. After removing low-quality reads without enzyme recognition sites, 66–76% of reads mapped uniquely to about 3 million enzyme recognition sites (Table 1).

**Figure 1.**
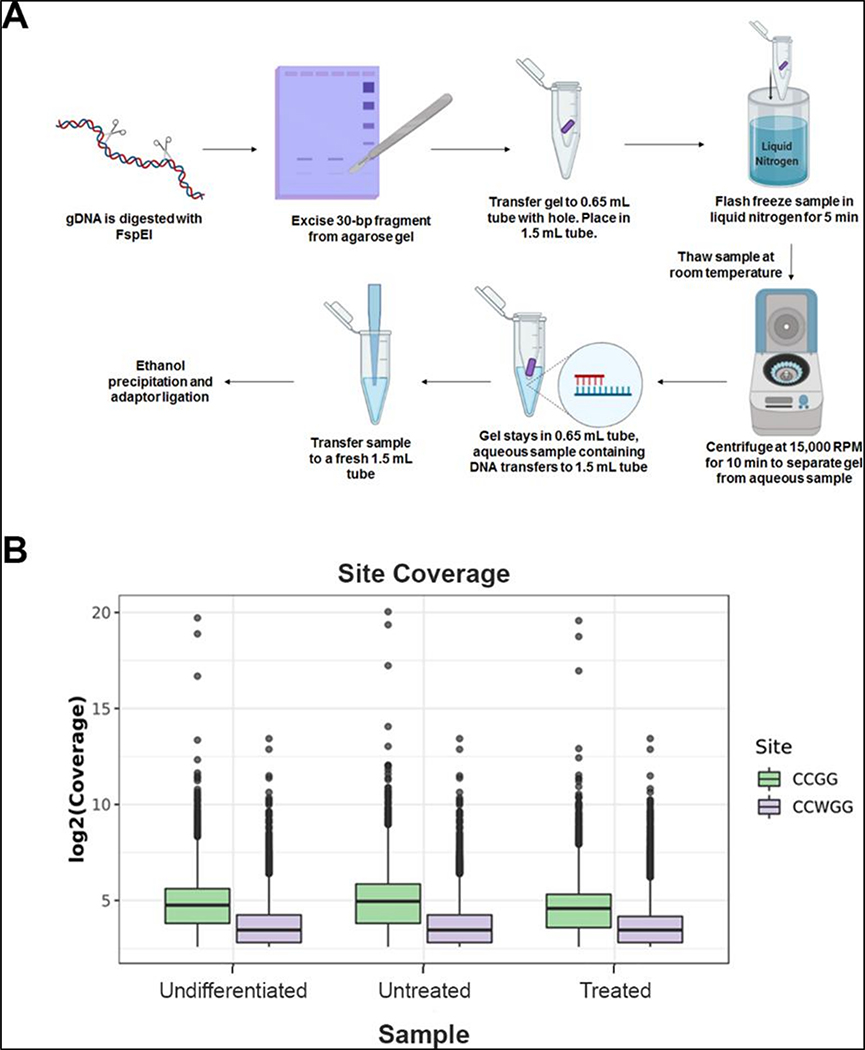
MethylRAD sequencing method and analysis: (**A**) flow chart illustrating stepwise protocol for genomic DNA digestion and purification of 31 to 32 bp fragments from agarose gel which are used for library preparation for high throughput sequencing. (**B**) Coverage of CCGG and CCWGG sites identified in each MethylRAD sample. Box plots showing the sequencing depths of the methylation sites (CCGG and CCWGG) in each sample of undifferentiated embryonic stem cells (Undifferentiated), day 7 differentiated cells (Untreated), and day 7 differentiated cells treated with pargyline (Treated). Green boxplots show the log2 coverage distribution for CCGG sites and purple boxes show the distribution for CCWGG (CCAGG and CCTGG sites). Center lines in the boxplots show the median, and boxes show the interquartile range (IQR), with the bottom of the boxes showing the first quartile (Q1), and the top of the box showing the third quartile (Q3). The bottom and top whiskers show Q1–1.5*IQR and Q3+1.5*IQR, respectively.

We determined the reliable methylated sites by using a cutoff of read coverage of no less than five reads for each site, and obtained 0.89 million CCGG sites and 1.21 million CCWGG sites on average for each sample. The average coverage depth of CCGG and CCWGG sites was 34.8 and 14.7, respectively. (Figure 1, Table 2).

To test the fidelity and coverage of our method, we compared it to bulk ESCs RRBS data [32]. In total, the RRBS data had 2,426,133 unique sites with read support of greater than five. Our MethylRAD data identified 3,212,889 unique sites with read support greater than five, leading to a 32% increase in the number of unique CpG sites detected (Table 3). Given the high fidelity of mapping and read number, we used the data for comparative analysis of DNA methylation between the samples.

### 2.2. Distribution of DNA Methylation Changes in ESCs during Differentiation

**Figure 2.**
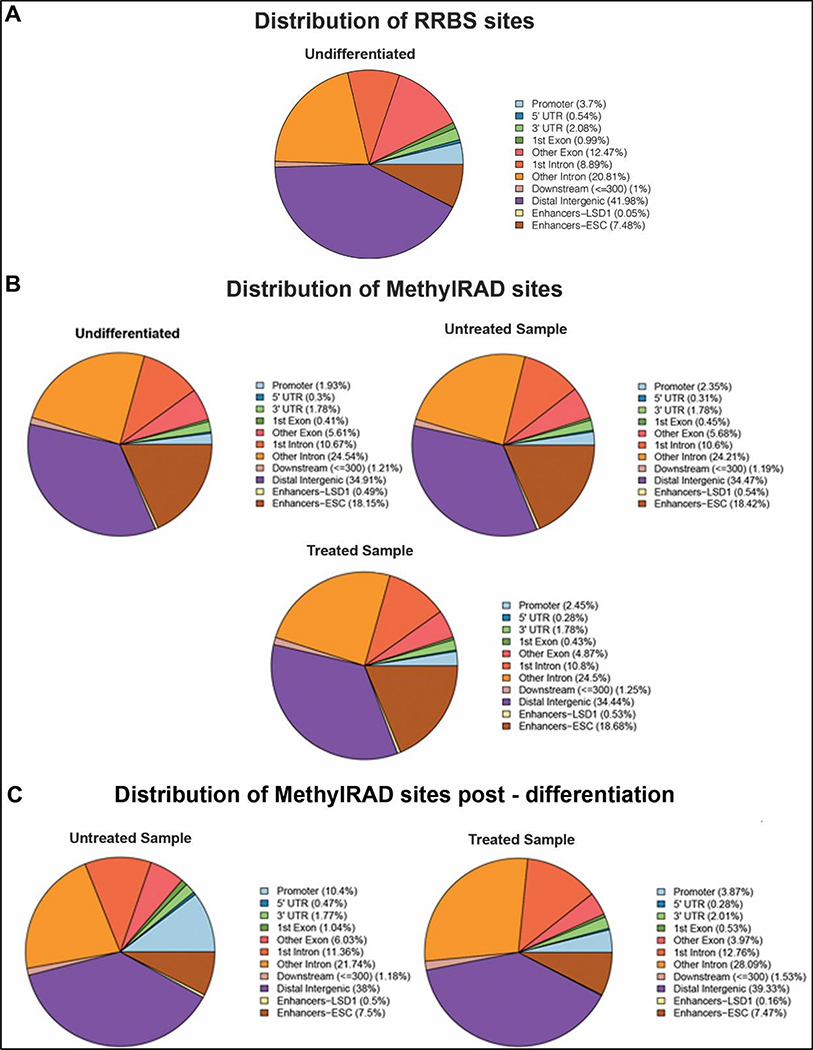
Distribution of methylated regions. Fractional distribution of mapped methylated sites was performed across regulatory regions of the genome. (**A**) The pie chart represents methylated sites identified in undifferentiated embryonic stem cells in RRBS data. (**B**) The pie charts represent methylated sites identified in MethylRAD data in embryonic stem cells (ESCs) in undifferentiated state, day 7 post differentiation (Untreated), and day 7 post differentiation treated with pargyline (Treated). (**C**) The pie charts show fractional distribution of sites identified by MethylRAD that are methylated only post differentiation in untreated or pargyline-treated cells.

We analyzed the distribution of the methylated sites from the RRBS data and the CCGG/CCWGG sites from our MethylRAD data in distinct genomic sequences in undifferentiated ESCs (Figure 2A,B). The genomic regions analyzed include 3′ UTRs, 5′ UTRs, exon, intron, intergenic, promoter, and enhancer regions. Most of the DNA methylation was detected in the distal intergenic regions, introns, and ESC-specific enhancers in both undifferentiated MethylRAD and RRBS sites (Figure 2A,B). Given that RRBS uses methylation-insensitive restriction enzymes, such as MspI (CCGG), which cut unmethylated CpG-rich regions, a higher proportion of promoters are detected with RRBS compared to MethylRAD. However, interestingly, there is a 10-fold increase in the LSD1-bound enhancers and about a 2-fold increase in ESC enhancers in MethylRAD data compared to RRBS. This can be explained by the “loose” specificity of the methylation-sensitive enzyme FspEI which can detect and cut around most methylated cytosines. These data support MethylRAD as an improved technique to detect methylated sites in CpG-poor regions, such as enhancers. We next analyzed the distribution of the methylated CCGG/CCWGG sites in distinct genomic sequences in differentiated cells in the absence and presence of the LSD1 inhibitor, pargyline (Figure 2B). Compared to the undifferentiated ESCs, the relative distribution of the percentage of methylation was similar between all three samples. Only a modest increase in DNA methylation was observed in promoter regions in the untreated (2.35%) and treated (2.45%) samples compared to the undifferentiated ESCs (1.93%), which most likely is associated with gene repression post differentiation [33].

We next focused on regions that were de novo methylated post differentiation and plotted the fractional distribution of methylation at these sites. A substantial gain of methylation was observed in promoters and enhancers (ESC and LSD1-bound) (Figure 2B). Interestingly, in pargyline-treated differentiated samples, this gain in methylation, particularly in promoters and LSD1-bound enhancers, showed 3–5-fold decrease in fractional amount. The 5′ UTR and exonic regions were also affected. Taken together, these data show the applicability of our MethylRAD analysis to detect changes in DNA methylation in a site-specific manner across the genome.

### 2.3. DNA Methylation in LSD1-Bound Enhancers is Affected by LSD1 Inhibition

We performed an in-depth analysis of changes in DNA methylation in the LSD1-bound PpGes by quantifying its increase or decrease in differentiated cells compared to undifferentiated ESCs. Our MethylRAD analysis detected methylation in 2316 of 3712 annotated LSD1-bound PpGes. To improve the statistical significance of our analysis, average methylation change in the exonic regions of housekeeping genes was used to normalize the data. Our data show a significant increase in methylation in about 27% enhancers post differentiation. LSD1 inhibition led to a significant reduction in the number of enhancers that gain methylation post differentiation (6.31%) (Figure 2.3A,B). Previous studies reported that of 3712 LSD1-bound PpG enhancers, 2738 (~74%) are decommissioned and undergo demethylation of H3K4me1 post differentiation. Consistent with the observations made for LSD1-bound enhancers, our data show that 29% of the decommissioned enhancers increase in DNA methylation, which was reduced to 7% in pargyline-treated cells (Figure S2A,B). Interestingly, a small fraction of PpGes showed a decrease in DNA methylation post differentiation, which increased in pargyline-treated samples. This could be due to a spurious low activity of DNMT3A in undifferentiated cells or during random differentiation events that is affected by pargyline treatment.

We have previously shown that the activity of LSD1 is inhibited by its interaction with the OCT4 transcription factor bound in PpGes. Ingenuity pathway analysis (IPA) further showed that LSD1-regulated PpGes were associated with OCT4-regulated genes [34]. Consistent with previous observations, the IPA of methylated PpGes showed that the associated genes are largely regulated by OCT4 and this enrichment was lost upon pargyline treatment (Figure 2.3C,D). These data support the role of OCT4/LSD1 activity in the regulation of DNA methylation in these enhancers.

### 2.4. Sparsely Methylated Enhancers Are Strongly Affected by LSD1 Inhibition

**Figure 4.**
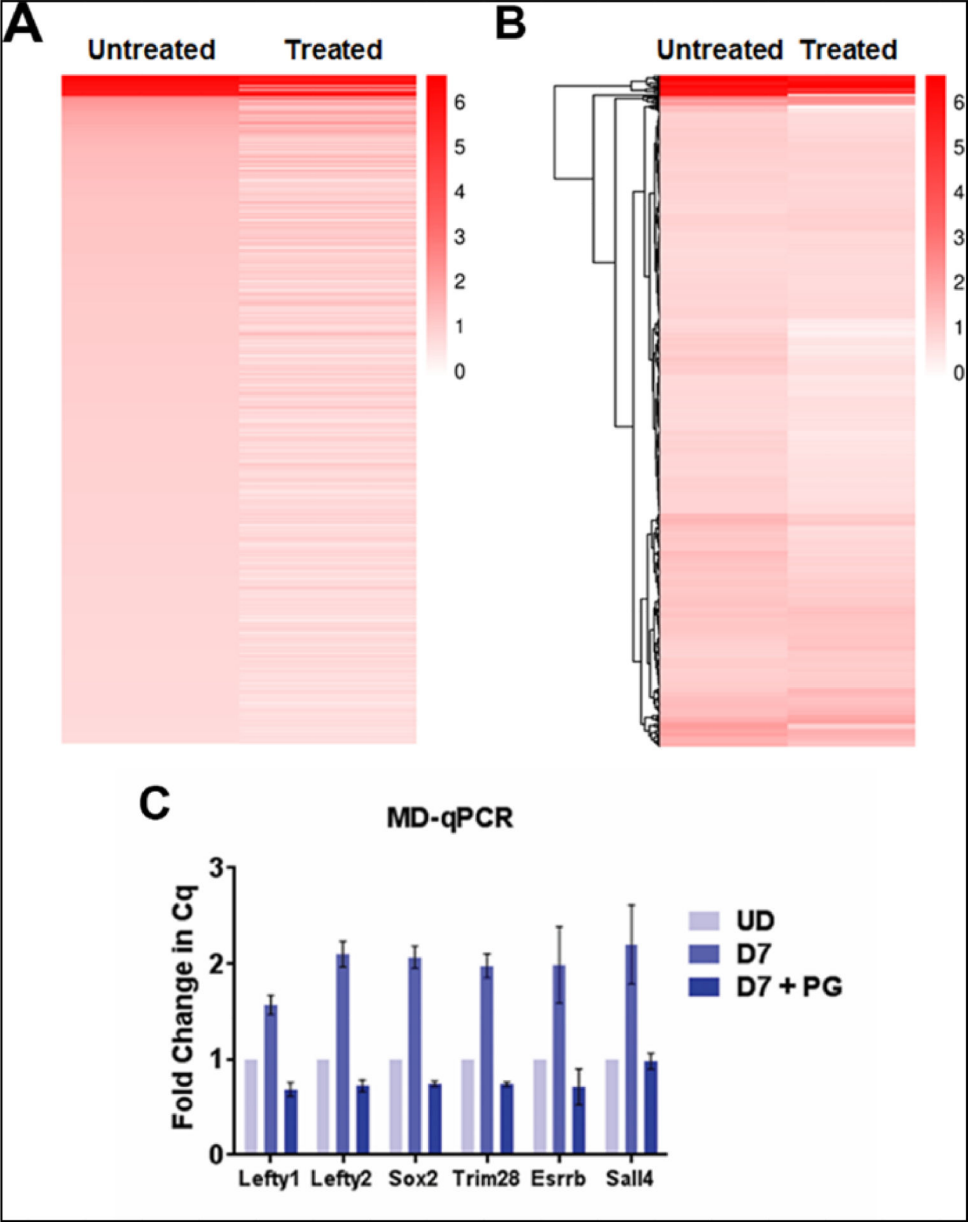
Heat map displaying the level of DNA methylation in LSD1-bound enhancers that gain methylation post ESC differentiation. Reads were quantified for each enhancer and the fold change of day 7 differentiated (untreated or pargyline-treated) to undifferentiated counts was log transformed. Light to dark red depicts a higher gain in DNA methylation post differentiation and white depicts little or no change. (**A**) Enhancers are ranked from greatest to smallest increase in methylation in untreated cells, whereas the treated panel compares the DNA methylation of these enhancers in pargyline-treated samples. A significant fraction of enhancers have lower DNA methylation [29] in treated cells compared to untreated. (**B**) Hierarchical clustering based on the Euclidean difference in log fold change is shown in a dendrogram. A clear pattern of enhancer hypomethylation is visible in the treated panel. (**C**) DNA methylation in listed enhancers was measured by methylation-dependent qPCR (MD-qPCR). The DNA was restricted using methylation-dependent enzyme MspJI, which cuts at methylated cytosines. The specific enhancers were amplified using the cleaved DNA as a template by qPCR. The Cq values for differentiated untreated and treated samples were normalized to those of undifferentiated samples. An increase in Cq value indicates a gain of DNA methylation, which was absent in pargyline-treated samples.

We next compared the impact of LSD1 inhibition on the gain of DNA methylation in PpGes by ranking from highest to lowest the increase in DNA methylation post differentiation and comparing DNA methylation in pargyline-treated samples. Interestingly, the heatmap shows that, whereas highly methylated enhancers were not affected by pargyline treatment, enhancers with a moderate level of methylation were hypomethylated (Figure 4A). We performed unsupervised hierarchical clustering to group the enhancers showing specific patterns. The data again confirm reduced methylation in pargyline-treated cells and an overall enhancer hypomethylation phenotype (Figure 4B). Indeed, when we performed DNA methylation analysis for individual PpGes through methylation-dependent qPCR (MD-qPCR), the gain in DNA methylation in the PpGes of *Lefty1, Lefty2, Sox2, Trim28, Esrrb*, and *Sall4* in untreated differentiated cells was strongly reduced in pargyline-treated differentiated cells. (Figure 4C). These data confirm that LSD1 inhibition has a widespread effect on the gain of DNA methylation in PpG enhancers post ESC differentiation.

### 2.5. Enhancers with Intermediate CpG Density Are Sensitive to LSD1 Inhibition

Most enhancers have been reported as regions with low/intermediate CpG content, making them susceptible to regulation by differential methylation [35,36]. However, some enhancers reside in CpG-rich regions, including CpG islands, which are hypermethylated in cancer and could be regulated by differential DNA methylation [37]. Given that our data in Figure 4A show a bimodal change in DNA methylation, where highly methylated enhancers are not affected by LSD1 inhibition, we asked if there is a correlation between the change in DNA methylation and the CpG content of the enhancers. We first determined the overlap of all MethylRAD sites with regions of high, intermediate, and low CpG content to determine the relative percentage in each group. For all three samples, more than 85% of sites were mapped to regions with intermediate CpG content and about 12–13% were mapped to regions with high CpG content (Figure S3A).

**Figure 5.**
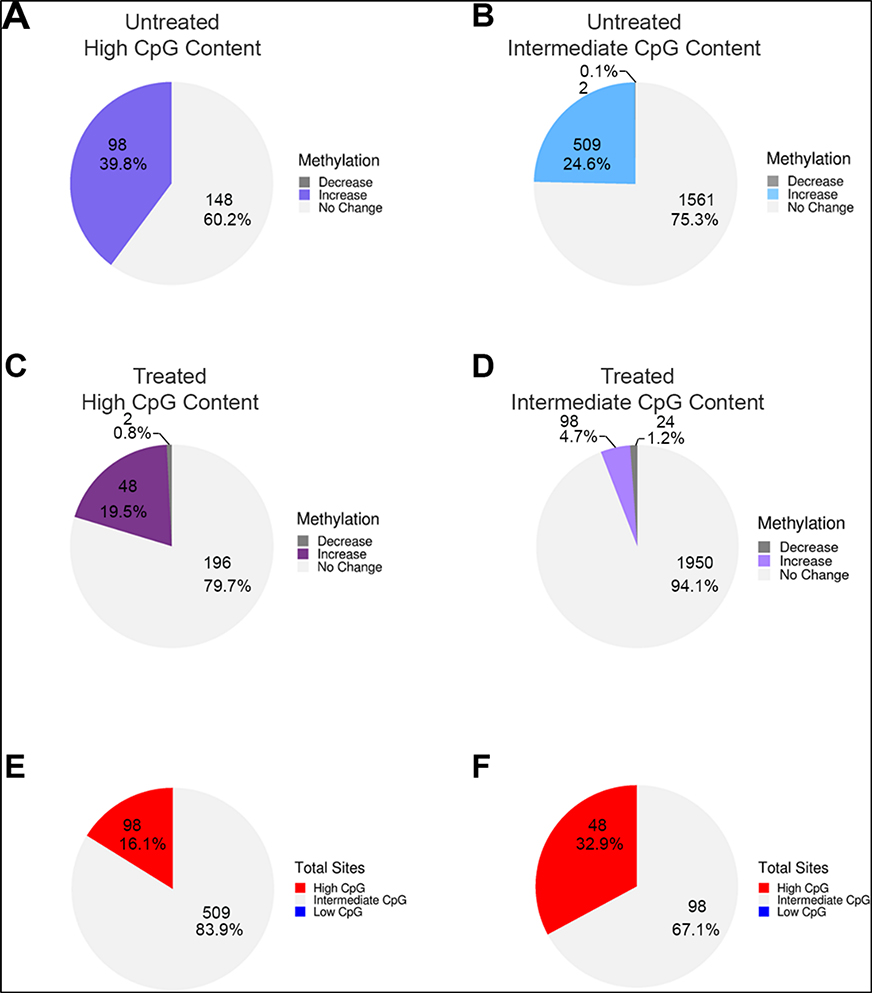
Frequency of DNA methylation correlated with CpG content of PpGes. LSD1-bound PpGes were sorted based on high and intermediate/low CpG content. Pie charts showing change in DNA methylation in untreated cells post differentiation in PpGes with (**A**) high CpG content and (**B**) intermediate/low CpG content, and in pargyline-treated cells post differentiation in PpGes with (**C**) high CpG content and (**D**) intermediate/low CpG content. The data show a 5-fold decrease in the number of PpGes that are methylated post differentiation in (**D**) compared to (**B**) and only a 2-fold decrease in (**C**) compared to (**A**). Direction of change (increase, no change, or decrease), was determined by normalizing to average methylation change in housekeeping genes. PpGes that get methylated post differentiation in the untreated and treated cells (shown in green and purple areas in the waterfall plot of Figure 2.3) were sorted based on their CpG content. Pie charts show CpG content of methylated PpGes post differentiation in untreated samples (**E**) and treated samples (**F**). DNA methylation gain in PpGes with high CpG content is more resistant to pargyline treatment.

We next sorted LSD1-bound enhancers with high and intermediate/low CpG content and determined the fractional distribution of change in DNA methylation during ESC differentiation in the absence and presence of pargyline. We detected a gain of DNA methylation post differentiation in ~40% of high-CpG content enhancers and 25% containing low/intermediate CpG content (Figure 5A,B). Interestingly, whereas the fraction of methylated high-CpG content enhancers was only reduced to 20% by pargyline treatment, the fraction of methylated low/intermediate-CpG content enhancers was reduced to ~5% (Figure 5C,D). These data suggest that LSD1-mediated regulation of DNA methylation is more prevalent in low/intermediate-CpG content enhancers. We tested this by determining the CpG content of the LSD1-bound enhancers that were decommissioned as defined by demethylation of H3K4me1 post differentiation (Figure S3B) [29]. The data showed that more than 90% decommissioned enhancers have intermediate CpG content, supporting this observation. It is possible that in CpG-rich enhancers, the presence of H3K4 trimethylation attracts other histone demethylases, such as Jumonji enzymes [38], making the gain of DNA methylation less sensitive to LSD1 inhibition. Supporting this prediction, our data showed that, post differentiation, only 16% of methylated PpGes have high CpG content, but in pargyline-treated differentiated samples, 33% have high CpG content (Figure 5E,F).

Taken together, our data describe the use of MethylRAD as a simple and sensitive method to determine changes in DNA methylation in PpGes during ESC differentiation. Our data show a widespread effect of LSD1 inhibition on the gain of DNA methylation in LSD1-bound PpGes. A significantly larger fraction of enhancers affected by LSD1 inhibition contain low/intermediate CpG content, suggesting that LSD1-mediated regulation of DNA methylation is potentially guided by CpG content.

## 3. Discussion

Previous studies have demonstrated the activity of the LSD1/Mi2/NuRD complex on the enhancers of PpGs [29]. In response to the signals of differentiation, the enzyme complex deacetylates and demethylates the histone H3 at K27 and K4, respectively. This is followed by activation of the DNA methyltransferase DNMT3A, which methylates the DNA at these sites [30,39]. This mechanism was shown in a subset of PpGes and is speculated to be widely prevalent in other PpGes. In this study, we used MethylRAD sequencing to identify PpGes which gain DNA methylation, which is dependent on H3K4me1/2 demethylation by LSD1 [23]. We first optimized the MethylRAD protocol to be technically straightforward and simple. The changes made in the method allowed for a higher recovery of restriction fragments by excising the 32 bp FspEI restriction fragments from an agarose gel which was purified and used for all the downstream processing. This ensured that the sequenced library contained FspEI sites. In contrast, in the original MethyRAD protocol, the whole genomic DNA after FsPEI digestion was treated for adapter ligation and amplification. This resulted in a mixture of DNA fragments which were separated on polyacrylamide gel and approximately a 100 bp DNA fragment was extracted by gel excision for library preparation and sequencing. This made the identification of the correct band tedious and the process irreproducible. The identification of this band from multiple fragments, which are very close in size, was very challenging and often resulted in the failure of the identification of FspEI sites post sequencing due to the retrieval of a wrong band. Our method, in which FspEI fragments are harvested in the beginning and exclusively used for downstream processing, ensured successful identification of methylated sites and enhanced coverage. Improved sensitivity allowed for regions with lower methylation to be recorded with higher accuracy, thus making it a more robust method to measure small changes in DNA methylation. Our data, which showed a more than 32% increase in CpG coverage compared to RRBS [32], further supported these improvements. Moreover, this tool, unlike other methods, can detect non-canonical cytosine methylation (CpA and CpT) with the same efficiency as for CpG methylation. However, given that the amount of CpG methylation is many times higher than CpA/CpT methylation, which cannot be maintained by DNMT1, it is challenging to accurately measure detectable changes in CpA methylation. Increasing the sequencing depth of the MethylRAD fragments will help improve the accuracy of this measurement. It is also important to note that RRBS gives information on methylated and on non-methylated DNA, allowing for a simpler deduction of the percent of methylation in any region, whereas MethylRAD only detects methylated DNA. However, it is possible to use the low read count or absence of reads at a site in MethylRAD data as a proxy for being unmethylated. Given that most differentially methylated regions, such as enhancers, often have a low “background” methylation even in their active state, we were able to detect these enhancers in the undifferentiated cells with low read counts (five reads minimum). An increase in methylation at these sites was observed by a substantial increase in the number of reads at these sites. The data suggest that the specificity and sensitivity of the MethylRAD assay is very suitable for CG-poor regions which are often differentially methylated, which is supported by a higher representation of enhancers in MethylRAD data compared to the RRBS data (Figure 2A,B).

In this study, we used ESC differentiation to study DNA methylation changes in specific regions of the genome. Given that our primary goal was to analyze methylation changes in LSD1-bound PpGes, we also differentiated ESCs in the presence of an LSD1 inhibitor, pargyline, and compared DNA methylation changes with those in untreated samples. Our data showed that the overall distribution of DNA methylation in various functional elements remains unchanged in undifferentiated and differentiated cells. However, there is a significant increase in DNA methylation in promoters and LSD1-bound PpGes in untreated differentiated cells compared to the pargyline-treated cells. These data emphasized the specificity of LSD1-mediated regulation of DNA methylation in enhancers and promoter regions of the genome. It also suggests that the loss of regulation of DNA methylation in LSD1-bound enhancers could impact the gain of DNA methylation in specific promoters. Given that promoters and enhancers interact in an enhancer–promoter (E-P) loop, we suggest a potential role for E-P loops in the regulation of promoter methylation, which could be experimentally tested. However, if pargyline treatment directly prevents gene repression, then decreased DNA methylation in promoters could also be a consequence of the continued transcription of these genes.

We next focused on DNA methylation changes in the LSD1-bound PpGes. Given that the restriction enzyme FspEI cuts methylated DNA, interestingly, we were able to detect about 53% of LSD1-bound enhancers, suggesting a low level of DNA methylation in these enhancers in the undifferentiated cells. This could be spurious methylation due to random differentiation in the tissue culture or actively maintained low methylation of unknown function. Although the number of enhancers detected post differentiation did not increase drastically (62%), a significant increase in the number of reads at the methylated enhancer sites was observed post differentiation (Table 1). The average depth of reads at CCGG sites also increased post differentiation (Table 2). The analysis revealed an increase in DNA methylation in about a quarter of these regions. The proportion of PpGes that gained methylation dropped to 6% in the presence of pargyline, demonstrating the extent of LSD1-mediated regulation of DNA methylation at these sites. A heatmap directly comparing methylated PpGes in treated and untreated cells showed a widespread hypomethylation in treated cells. The spatiotemporal regulation of DNA methylation in PpGes was shown in previous work to involve the role of transcription factor OCT4, which interacts with LSD1 and inhibits its activity [34,40–42]. The dissociation of OCT4 from PpGes allows for LSD1 to demethylate H3K4me1 in PpGes post differentiation, suggesting that PpGes that gain DNA methylation in an LSD1-dependent manner are potentially regulated by OCT4. This was confirmed by the enrichment of OCT4-dependent genes in pathway analysis of the methylated PpGes that is decreased in pargyline-treated cells.

In alignment with previously published data showing that DNA methylation changes are prevalent in functional elements with low/intermediate CpG content [9], our data showed that methylated PpGes have an overrepresentation of intermediate CpG content. However, interestingly, whereas a larger fraction of PpGes with high CpG content (39.8%) gained methylation compared to those with intermediate CpG content (24.6%), DNA methylation of PpGes with intermediate CpG content was more susceptible to pargyline treatment. These data suggested that LSD1-mediated regulation of DNA methylation prefers PpGes with intermediate CpG content. Although this relationship could be regulated by various mechanisms, we suggest that CpG-rich PpGes recruit other histone demethylases, making the gain of DNA methylation in these regions less sensitive to LSD1 inhibition.

In summary, we optimized the MethylRAD sequencing protocol and analysis tool for higher sensitivity and accuracy. Given that DNA methylation changes during disease and development occur in CpG-poor regions with lower DNA methylation, the ease and accuracy of this method can be exploited for rapid identification of differentially methylated regions.

## 4. Materials and Method

### 4.1. ESC Culture and Differentiation Method

E14Tg2A (WT) ESCs were maintained in media containing leukemia inhibitory factor (LIF) and induced to differentiate by LIF withdrawal followed by retinoic acid addition. Treatment with the LSD1 inhibitor pargyline (3mM) (Sigma P8013) (Millipore, 616431, Burlington, Massachusetts, USA) was performed as previously described [30]. For all experiments, this procedure was repeated at least two times for sample collection.

### 4.2. MethylRAD Library Preparation and Sequencing

Genomic DNA was isolated using standard phenol:chloroform extraction, followed by ethanol precipitation. DNA from various samples was digested with FspEI for 4 h at 37°C and subjected to electrophoresis in a 2% agarose gel. FspEI recognizes C^m^C and makes double-stranded breaks at the N_12_/N_16_ position from the modified cytosine. At CpG sites, FspEI cleaves bidirectionally, releasing 31 or 32 bp fragments. The DNA around 30 bp was excised out of the agarose gel and purified using a modified freeze and squeeze technique (Figure S1A) [43]. In brief, the excised gel pieces were placed inside a 0.65 mL micro centrifuge tube with a small hole in the bottom of the tube which was made using a 20-gauge needle. The 0.65 mL tube containing the gel piece was then placed in a 1.5 mL micro centrifuge tube and flash frozen in liquid nitrogen. The samples were then thawed at room temperature and centrifuged at 15,000 rpm for 10 min. This extruded the DNA along with the buffer into the 1.5 mL tube, leaving behind agarose in the small tube. The DNA was extracted by ethanol precipitation, resuspended in TE and quantified by a NanoDrop. This DNA was further used for adaptor ligation at 4°C overnight [23]. The ligated DNA was PCR amplified with index primers, purified using a QIAquick PCR purification kit and sequenced using a Novaseq 6000. The primers used for PCR amplification are listed in S1.

### 4.3. Alignment and Quality Control

Single-end 1 × 150 sequencing was performed on a NovaSeq 6000 for undifferentiated ESCs and day 7 differentiated samples. Base quality values were calculated using Phred score. The program FastQC v. 0.11.7 [44] was used to check data quality before and after quality trimming/adapter removal. Adapters were removed from reads using Trimmomatic v. 0.36 [45]. Trimmomatic is a program that removes adapter sequences and trims short Illumina reads based on quality. Cutadapt version 2.2 [46] was used to trim reads further, removing the first two and last two bases of each read. Reads containing more than 0 Ns were discarded, and low-quality (Phred score < 20) reads were removed. Reads which did not have FspEI sites present anywhere in the read were removed using “grep”. Finally, Bowtie2 version 2.3.3 [47,48] was used to map reads to the ENSEMBL *Mus musculus* reference genome version GRCm38.93. A maximum of one mismatch was allowed in read mapping. Reads mapped to the genome exactly one time were included in further analyses as it was not possible to judge the actual position of the methylation site in multi-mapped reads.

### 4.4. Methylation Site Identification and Quantification

Methylated sites were catalogued by iterating through all read sequences to find a matched pattern of CCGG/CCWGG methylation sites (i.e., CCGG, CCAGG, and CCTGG) and recording their location in the genome. The number of reads mapping to each methylated site was recorded and adjusted for substitution, deletion, and insertion accordingly. Sites were also matched with the reference genome for verification. Sites that had fewer than five reads were removed from downstream analysis as these were less reliable; counts from duplicate sites between patterns were summed as one site. The observed sequencing depth in MethylRAD data directly corresponded to the degree of methylation at the site.

### 4.5. Comparing MethylRAD Data to RRBS

MethylRAD data was compared to bulk RRBS data from Guo et al. [32]. BED files were downloaded from the Gene Expression Omnibus [GEO; http://www.ncbi.nlm.nih.gov/geo/], accession number GSE47343. BED file mm9 regions were converted to mm10 coordinates using CrossMap, and regions were then filtered to only include locations with at least five methylated reads in each dataset. Finally, overlap was found between MethylRAD and RRBS sites using BEDTools intersect v2.27.1. In quantifying the overlap between total sites identified in RRBS and MethylRAD, only unique sites were considered, and sites identified in multiple MethylRAD samples were only counted once per site.

### 4.6. Annotation to LSD1-Bound Enhancers

Sites in the LSD1-bound enhancers were modified to include 1 kb up- and downstream of the start site. All samples were annotated to the modified LSD1 regions using BEDTools intersect. The total methylation of a region was determined by combining the read counts of all sites in that region. Upper and lower quartiles were used in thresholding regions to define gaining or losing methylation. Specifically, all regions with more than 22 read counts in undifferentiated than in day 7 samples were identified as losing DNA methylation during differentiation. All regions with more than 30 read counts in day 7 samples than in undifferentiated samples were identified as gaining DNA methylation. Regions were divided into low, intermediate, and high methylation regions by comparing the level of methylation of LSD1-bound enhancers identified as methylated in the MethylRAD data to the level of methylation observed in all identified enhancers genome wide. First, the average length of enhancers in the EnhancerAtlas database (version 2.0) was calculated for each gene (if different studies had different lengths for the enhancers then these lengths were averaged). The LSD1-bound regions identified were always 2500 in length (500 bases from Whyte et al., X +1kb up- and downstream)]. The raw counts for each enhancer in each sample were then normalized by dividing by the length and then multiplied by 1000. Quartiles were calculated and used to determine regions of low (0, *Q*_1_), intermediate (*Q*_1_, *Q*_4_), and high (*Q*_4_, ∞) methylation.

### 4.7. Comparison of Methylation Levels between Samples

The union of enhancers found in both the undifferentiated 7 day and pargyline (PG)-treated ESC samples was determined. The differences in methylation for each combination of enhancer regions were computed by subtracting the undifferentiated sample counts from those of the other samples. Comparative figures were produced from these final data. Quartiles were used to determine the regions with the greatest loss of methylation and greatest gain of methylation between differentiated and undifferentiated samples. The greatest decrease in methylation was determined from untreated differentiated samples minus untreated undifferentiated samples to be (−∞, *Q*_1_), no significant change was between (*Q*_1_, *Q*_4_), and the greatest increase in methylation was (*Q*_4_, ∞).

### 4.8. Calculating CpG Content

The genome was divided into 500 bp windows with five bases of overlap. In each window, the length of the window, as well as the number of Cs, Gs, and CpGs, were calculated. For each window, CpG content was calculated as previously described [49]. CpG ratio = (number of CpGs x number of bps)/(number of Cs x number of Gs). The three categories of CpG content were classified as follows: high CpG regions are windows with a CpG ratio above 0.75 and a GC content above 55%, low CpG regions are windows with a CpG ratio below 0.48, and intermediate CpG regions are all other windows. The MethylRAD sites were overlapped with regions of high, intermediate, and low CpG content to determine the percentage of all sites which were found in high-, intermediate-, and low-CpG content windows.

### 4.9. Pathway Analysis

Differentially methylated sites were associated with genes and were used to perform a pathway analysis and a functional enrichment analysis. Pathway analyses were performed on LSD1 regions divided by degree of methylation (low, intermediate, and high methylation), as well as on regions with a significant decrease in methylation, increase in methylation, and no change in methylation between differentiated and undifferentiated samples. Ingenuity Pathway Analysis software (IPA, QIAGEN Redwood City, www.qiagen.com/ingenuity) was used in the annotation of genes and in performing the pathway analyses. All p-values were corrected for multiple testing using the Benjamini–Hochberg method. Pathways and functional categories were enriched if the adjusted *p*-value < 0.05.

### 4.10. DNA Methylation-Dependent qPCR Assay (MD-qPCR)

Genomic DNA was harvested using a standard phenol:chloroform isolation, followed by ethanol precipitation. DNA from samples was digested first with CviQI (NEB, R0639L) overnight at 25°C. Purified samples were then digested with FspEI (NEB) overnight at 37°C. The digested DNA was purified and quantified by PicoGreen according to the manufacturer’s protocol (Life Technologies, P11495) using a NanoDrop 3300 fluorospectrometer. Quantitative PCR was performed using an equal amount of DNA for each sample. The change in DNA methylation is represented by relative fold change in the Cq value as follows: 2ˆ(Cq(U)-Cq(I)), where Cq(U) is the Cq for the undifferentiated ESC sample, and Cq(I) represents treated or untreated day 7 differentiated ESCs. Cq is the quantification cycle as calculated by Biorad CFX Manager 3.1 based on the Minimum Information for Publication of Quantitative Real-Time PCR Experiments (MIQE) guidelines [50]. The primers used for DNA methylation-dependent qPCR analysis have been previously described [30]. Standard deviations represent three technical and two biological replicates.

## Supplementary Material

Supplementary

## Figures and Tables

**Figure 3.**
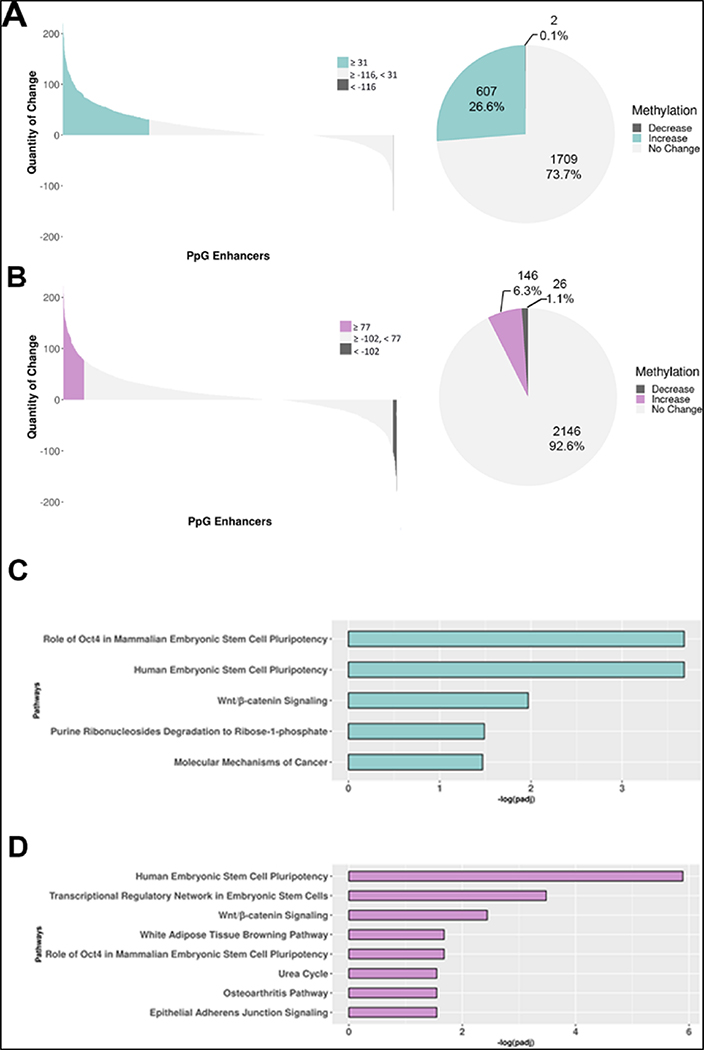
DNA methylation change in LSD1-bound pluripotency gene enhancers during ESC differentiation. (**A**,**B**) Genome-wide DNA methylation analysis by MethylRAD sequencing. The number of MethylRAD reads per LSD1-bound pluripotency gene enhancer (PpGe) region were used as a measure for the extent of DNA methylation and compared between day 7 (D7) differentiated (untreated or treated) and undifferentiated (UD) samples. Untreated (**A**); treated (**B**). The left panel of the waterfall plot shows DNA methylation changes in pluripotency gene enhancers (PpGes), which were computed by subtracting normalized read counts in D7 differentiated samples from normalized counts in UD samples. Upper and lower quartiles were used in thresholding regions as gaining or losing methylation. The right panel of the pie chart shows fractional distribution of PpGes with an increase, decrease, or no change in DNA methylation. (**C,D**) Statistically significant enriched canonical pathways among the genes associated with increased DNA methylation in enhancers. (**C**) Pathways that had higher enhancer DNA methylation in D7 untreated differentiated samples than UD. (**D**) Pathways that had higher enhancer DNA methylation in pargyline-treated D7 differentiated samples than in UD. The *x*-axis shows the log_10_ (adjusted p-value), with the p-values adjusted for multiple testing using the Benjamini–Hochberg method.

**Table 1.** Read statistics and mapping. Sample sequencing data quantity versus ratio [the ratio of enzyme reads to the unique position of the reference sequence is shown] in undifferentiated embryonic stem cells (UD), day 7 differentiated cells (Untreated), day 7 differentiated cells treated with pargyline (Treated).

Samples	Total Reads	Clean Data	Reads with FspEI Site	Reads Mapped	% Uniquely Mapped Reads
UD	128,245,179	128,244,535	67,257,685	50,959,931	76%
Untreated	191,444,768	191,547,179	103,231,509	73,712,891	71%
Treated	184,231,969	184,230,068	101,304,461	67,235,311	66%

**Table 2.** Sites statistics and mapping. The average sequencing depth of the methylation site (CCGG and CCWGG) in undifferentiated embryonic stem cells (UD), day 7 differentiated cells (Untreated), day 7 differentiated cells treated with pargyline (Treated).

	CCGG		CCWGG	
Sample	# Sites	Average Depth	# Sites	Average Depth
UD	870,345	36.29	538,222	15.96
Untreated	882,955	41.48	1,313,633	15.24
Treated	892,366	28.84	1,162,815	14.00

**Table 3.** Overlap between MethylRAD and reduced representation bisulfite sequencing (RRBS) data. In each dataset, only regions which had at least five methylation events were considered. Percent RRBS overlap shows the percentage of sites in RRBS data that are also captured by the MethylRAD data. Percent MethylRAD overlap shows the percentage of sites in MethylRAD data that are also captured by the RRBS data.

Sample	Sites Intersecting with RRBS (>5 Reads per Site)	Percent MethylRAD Overlap	Percent RRBS Overlap
UD	126625	9%	20%
Untreated	130904	9%	21%
Treated	138419	7%	22%
All MethylRAD	164345	5%	26%
