# Supplementary material for: Simplified MethylRAD Sequencing to Detect Changes in DNA Methylation at Enhancer Elements in Differentiating Embryonic Stem Cells
Source: Epigenomes. 2020 Oct 1;4(4):24. doi: 10.3390/epigenomes4040024 (PMC8023688; doi:10.3390/epigenomes4040024)
Supplement: Supplementary file 1 [file epigenomes-04-00024-s001.pdf]

epigenomes

Simplified MethylRAD Sequencing to Detect Changes in DNA Methylation at Enhancer Elements in Differentiating Embryonic Stem Cells

Debapriya Saha[1], Allison B Norvil[1], Nadia A. Lanman[2,3] and Humaira Gowher[1,2*]

[1]Department of Biochemistry; [2]Purdue University Center for Cancer Research; [3]Department of Comparative Pathobiology; Purdue University; West Lafayette, Indiana 47907 USA
*Correspondence: hgowher@purdue.edu

Keywords: DNA methylation; embryonic stem cells; MethylRAD; enhancer; pluripotency genes; histone demethylase LSD1

Supplementary Figures and Table

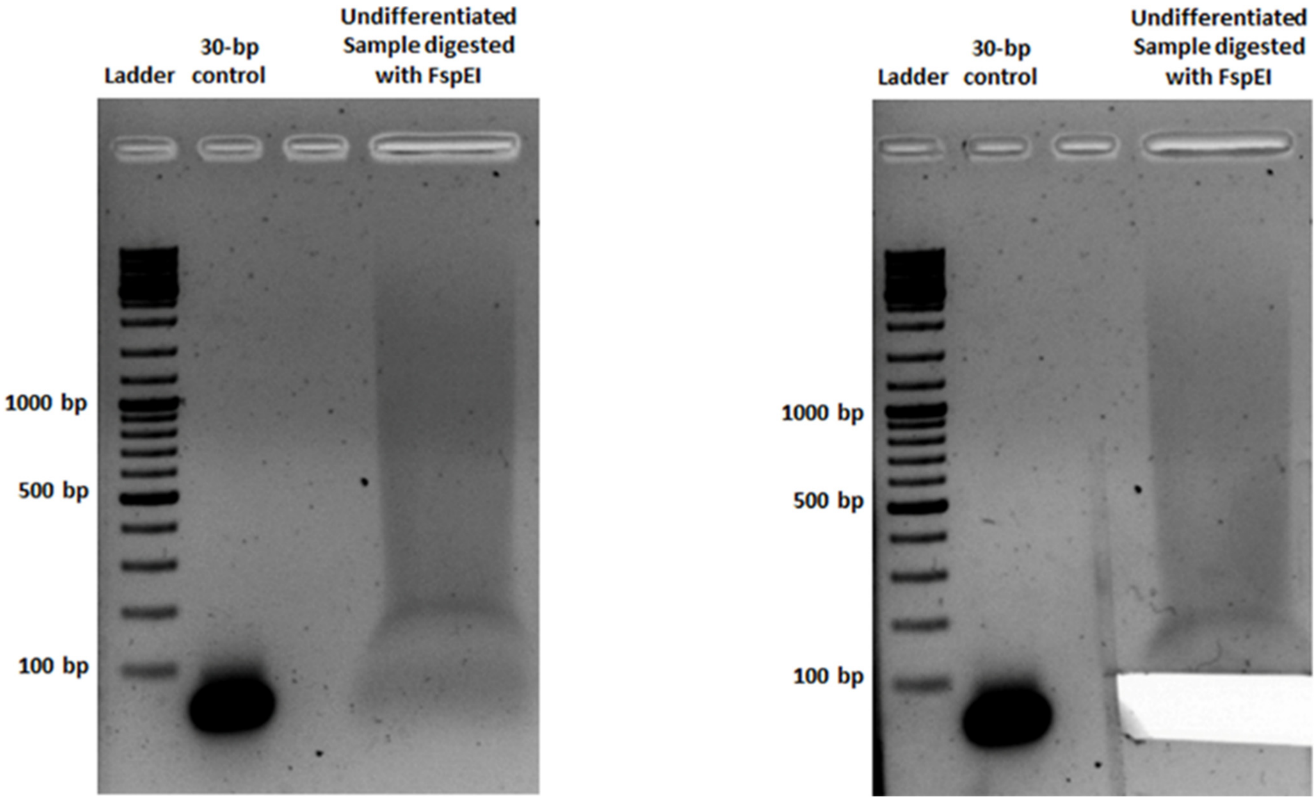

Supplementary Figure S1. Example of agarose gel electrophoresis of 30-bp control and FspEI digested gDNA. Left Original agarose gel with both samples. Right Agarose gel with 30-bp region excised from the FspEI digest gDNA sample.

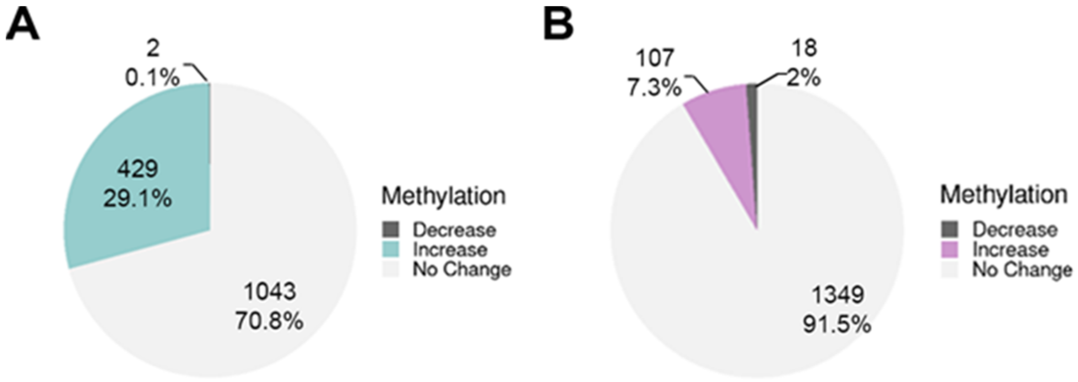

Supplementary Figure S2. DNA methylation changes at decommissioned PpGe: Post differentiation, many LSD1 bound PpGe get demethylated at H3K4me1. Pie chart shows fractional distribution of these decommissioned PpGe across increase, decrease, or no change in DNA methylation state in differentiated untreated (**A**) and pargyline treated (**B**) samples.

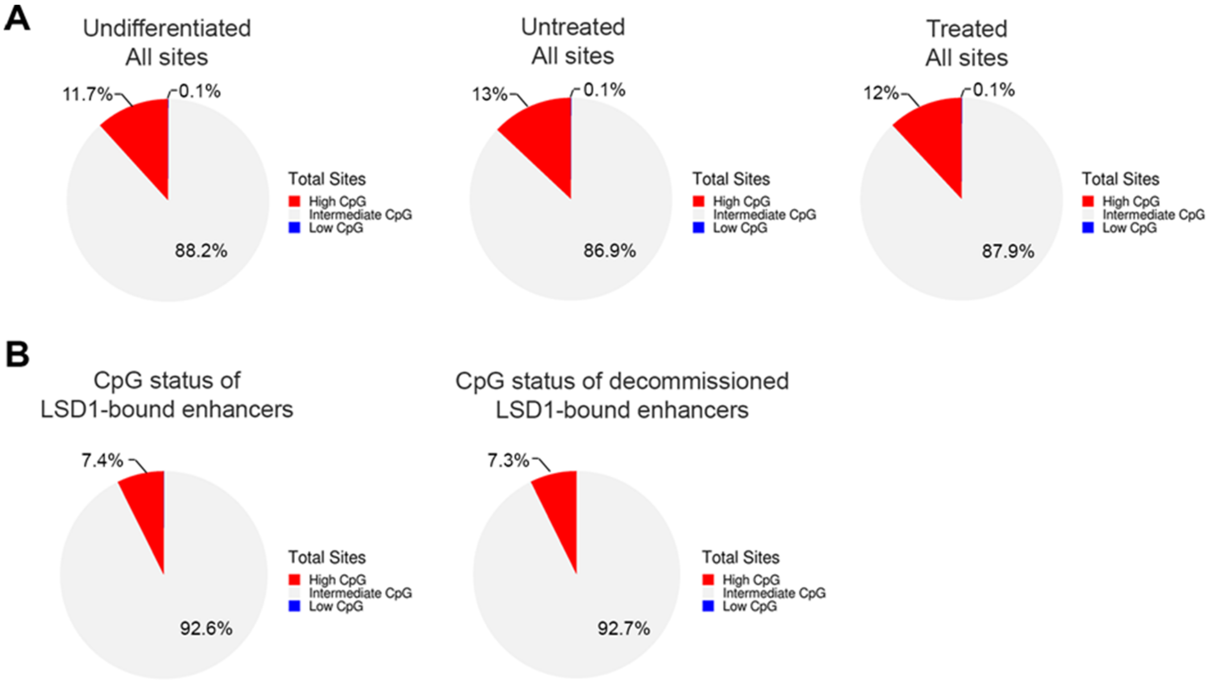

Supplementary Figure S3. (**A**) Pie charts show CpG content of all the MethylRAD sites in undifferentiated and post differentiation in untreated samples and treated sample. (**B**) Pie chart showing CpG content distribution of Lsd1 bound and decommissioned pluripotency gene enhancers.

Supplementary Table S1. Adaptors and primers used for MethylRAD library preparation. Oligonucleotide sequence of adaptors and primers for MethylRAD technique. The 3′ end of the antisense nucleotide sequence of each adaptor is blocked with an amine group to prevent extension.

MethylRAD-seq adaptor and index primers	
Adaptor-1 sense	ACACTCTTTCCCTACACGACGCTCTTCCGATCT
Adaptor-1 antisense	NNNNAGATCGGAAGAGC(AminoC6)
Adaptor-2 sense	GTGACTGGAGTTCAGACGTGTGCTCTTCCGATCT
Adaptor-2 antisense	NNNNAGATCGGAAGAGC(AminoC6)
P1	ACACTCTTTCCCTACACGACGCT
P2	GTGACTGGAGTTCAGACGTGTGCT
i5- UDA5073	AATGATACGGCGACCACCGAGATCTACACGATAACAAGTACACTCTTTCCCTACACGACGCT

i5- UDA5074	AATGATACGGCGACCACCGAGATCTACACAGCGGTGGACACACTCT TTCCCTACACGACGCT
i5- UDA5075	AATGATACGGCGACCACCGAGATCTACACGGTTATGCTAACACTCTT TCCCTACACGACGCT
i5- UDA5076	AATGATACGGCGACCACCGAGATCTACACAACCGCATCGACACTCT TTCCCTACACGACGCT
i7- UDA7120	CAAGCAGAAGACGGCATACGAGATGAACTTCCTTGTGACTGGAGTT CAGACGTGT
i7- UDA7119	CAAGCAGAAGACGGCATACGAGATAGGTCCTTCCGTGACTGGAGTT CAGACGTGT
i7- UDA7118	CAAGCAGAAGACGGCATACGAGATTATGGAGATTGTGACTGGAGTT CAGACGTGT
i7- UDA7117	CAAGCAGAAGACGGCATACGAGATCGCAAGAGCCGTGACTGGAGTT CAGACGTGT
